# Investigation of Upper Respiratory Carriage of Bacterial Pathogens among University Students in Kampar, Malaysia

**DOI:** 10.3390/tropicalmed8050269

**Published:** 2023-05-08

**Authors:** Hing Huat Ong, Wai Keat Toh, Li Ying Thong, Lee Quen Phoon, Stuart C. Clarke, Eddy Seong Guan Cheah

**Affiliations:** 1 Department of Biological Science, Faculty of Science, Kampar Campus, Universiti Tunku Abdul Rahman, Kampar 31900, Malaysia; huat0207@1utar.my (H.H.O.); keiferwk@1utar.my (W.K.T.); thongliying97@gmail.com (L.Y.T.); 2 Department of Allied Health Sciences, Faculty of Science, Kampar Campus, Universiti Tunku Abdul Rahman, Kampar 31900, Malaysia; phoonlq@utar.edu.my; 3 Centre for Biomedical and Nutrition Research, Kampar Campus, Universiti Tunku Abdul Rahman, Kampar 31900, Malaysia; 4 Faculty of Medicine and Institute for Life Sciences, University of Southampton, Southampton SO16 6YD, UK; s.c.clarke@soton.ac.uk; 5 NIHR Southampton Biomedical Research Centre, University Hospital Southampton NHS Trust, Southampton SO16 6YD, UK; 6 Global Health Research Institute, University of Southampton, Southampton SO17 1BJ, UK; 7 School of Postgraduate Studies, International Medical University, Kuala Lumpur 57000, Malaysia; 8 Centre for Translational Research, Institute for Research, Development, and Innovation, International Medical University, Kuala Lumpur 57000, Malaysia

**Keywords:** upper respiratory tract, Malaysia, carriage study, *Streptococcus pneumoniae*, *Haemophilus influenzae*, *Neisseria meningitidis*, *Staphylococcus aureus*, *Klebsiella pneumoniae*, *Pseudomonas aeruginosa*

## Abstract

The carriage of bacterial pathogens in the human upper respiratory tract (URT) is associated with a risk of invasive respiratory tract infections, but the related epidemiological information on this at the population level is scarce in Malaysia. This study aimed to investigate the URT carriage of *Streptococcus pneumoniae*, *Haemophilus influenzae*, *Neisseria meningitidis*, *Staphylococcus aureus*, *Klebsiella pneumoniae* and *Pseudomonas aeruginosa* among 100 university students by nasal and oropharyngeal swabbing. The presence of *S. aureus*, *K. pneumoniae* and *P. aeruginosa* was assessed via swab culture on selective media and PCR on the resulting isolates. For *S. pneumoniae*, *H. influenzae* and *N. meningitidis*, their presence was assessed via multiplex PCR on the total DNA extracts from chocolate agar cultures. The carriage prevalence of *H. influenzae*, *S. aureus, S. pneumoniae*, *K. pneumoniae, N. meningitidis* and *P. aeruginosa* among the subjects was 36%, 27%, 15%, 11%, 5% and 1%, respectively, by these approaches. Their carriage was significantly higher in males compared to females overall. The *S. aureus*, *K. pneumoniae* and *P. aeruginosa* isolates were also screened by the Kirby-Bauer assay, in which 51.6% of *S. aureus* were penicillin-resistant. The outcomes from carriage studies are expected to contribute to informing infectious disease control policies and guidelines.

## 1. Introduction

*Streptococcus pneumoniae*, *Haemophilus influenzae*, *Neisseria meningitidis*, *Staphylococcus aureus*, *Klebsiella pneumoniae* and *Pseudomonas aeruginosa* are among the bacterial pathogens that colonize the human upper respiratory tract (URT) [1,2,3]. Their pathogenicity is mediated by some major virulence factors involved in host cell adherence and invasion, tissue damage and immune evasion [4,5,6,7]. Despite being pathogenic, most individuals do not show any symptoms when these bacteria colonize the URT. Under certain conditions, some of them (e.g., *S. pneumoniae*) can translocate to other URT sites causing local infections, such as otitis media and sinusitis, or to the lungs, bloodstream and brain, causing potentially life-threatening diseases, such as pneumonia, septicemia and meningitis, respectively [8,9,10,11,12]. One study reported that high-density colonization of the URT by *S. pneumoniae*, *H. influenzae* and *Klebsiella* spp. was associated with increased risks of lower respiratory tract infections [11]. This indicates that asymptomatic colonization (i.e., carriage) is the first step in developing respiratory tract infections [13].

The microbial communities in the URT vary significantly among different sites [2,11]. With regard to the target bacteria in this study, the microbiome of anterior nares is predominated by *S. aureus* [14], while the oropharynx commonly shows the presence of *S. pneumoniae* and *H. influenzae* [15]. Some bacterial pathogens carried in the URT, for instance *S. pneumoniae* and *N. meningitidis*, can be spread via airborne respiratory droplets, leading to colonization and possibly infection in the new hosts. This is especially apparent in crowded environments and among household contacts [15,16,17]. Hence, a higher prevalence of carriage might increase the risk of infection or outbreak within a population, particularly among the age extremes [18,19].

Respiratory carriage studies are a pragmatic solution to getting real-time epidemiological data on the carriage of pathogens at the population level [19]. Several carriage studies have been conducted among university students (representing young adults). A study of this type in Nepal reported that 35% and 12.5% of nasal and pharyngeal isolates were of *S. aureus* and *H. influenzae*, respectively [20]. A review of *N. meningitidis* carriage studies among university students in various countries revealed the highest carriage in the Americas (4–71.1%) and European region (10.4–61.9%), followed by Nigeria (5.1%), Chile (4%) and India (1.5%) [21]. In Malaysia, existing studies among university students mainly focused on *S. aureus*, with carriage prevalence from 10% to as high as 93% recorded [22,23,24]. Other carriage studies emphasized on pneumococcal carriage in children, in which the prevalence of about 10% was documented based on nasopharyngeal swab assessment [25,26]. Recent carriage studies were carried out among the indigenous communities (locally known as Orang Asli) in certain parts of Malaysia, in which high carriage of *S. aureus* (47.2%) and *K. pneumoniae* (30%) was observed [27,28].

To date, respiratory carriage studies are still limited in Malaysia, in which those reported previously are outdated and were skewed toward certain age and ethnic groups [27,28,29]. Further epidemiological studies are needed to increase the data on pathogenic bacterial carriage, especially among young adults and ethnic minority groups. Therefore, this study set out to investigate the URT carriage of *S. pneumoniae*, *H. influenzae*, *N. meningitidis*, *S. aureus*, *K. pneumoniae* and *P. aeruginosa*, focusing on students at a private university. The objectives were to determine the carriage prevalence of these bacteria and preliminarily assess their association with several host factors to provide a better understanding of the risk of disease acquisition from their carriage. The antibiotic susceptibility of isolates of the three ESKAPE pathogens (*S. aureus*, *K. pneumoniae* and *P. aeruginosa*) was also evaluated; these pathogens are commonly associated with high antibiotic resistance.

## 2. Materials and Methods

### 2.1. Study Population, Design and Procedures

The total number of subjects (*n* = 100) were randomly recruited from the student population at Universiti Tunku Abdul Rahman (UTAR), Kampar, located in the state of Perak, which lies on the west coast of Peninsular Malaysia. Ethical approval for the study was obtained from the UTAR Scientific and Ethical Review Committee (approval no. U/SERC/49/2018). The sample collection was carried out during the period of 1 May to 31 August 2018.

The sample size required was calculated using the Epi Info™ 7.2.2.6 software [30] available at the Centers for Disease Control and Prevention (CDC) website. The UTAR Kampar Campus (study location) saw the enrolment of approximately 13,280 students during the study period. Based on this population size and the expected frequency of 10% reported for pneumococcal carriage in a Malaysian study [28], the sample size of *n* = 95 was determined to be sufficient to attain a statistical power of at least 95% confidence level, with a margin of error of 6%.

Informed consent was obtained from all participants prior to sample collection. The information sheet provided to participants includes guidelines on confidentiality and anonymity of the data obtained. A questionnaire to collect socio-demographic and health-related data was completed for each subject, including the subject’s gender, age, ethnicity, vaccination status, self-reported respiratory symptoms and antibiotic consumption in the previous month. A nasal (anterior nares) swab and an oropharyngeal swab were taken from each subject by using sterile cotton swabs of the DRYSWAB™ range (MWE, Wiltshire, UK) in accordance with the WHO Pneumococcal Carriage Working Group guidelines [31]. The swab was placed in the skim milk, tryptone, glucose and glycerol (STGG) medium for laboratory analysis and long-term storage.

### 2.2. Swab Culture for Target Bacteria

The swab suspensions obtained were cultured onto general chocolate agar, mannitol salt agar (for isolation of *S. aureus*), MacConkey agar (*K. pneumoniae*) and Pseudomonas agar supplemented with cetrimide and sodium nalidixate (C-N) (*P. aeruginosa*). All the components for preparing these media were sourced from Oxoid (Hampshire, UK). The chocolate agar plates were incubated at 37 °C in a candle jar, while the other culture media were incubated at 37 °C under aerobic conditions. All the plates were incubated for 1–3 d. Preliminary identification of the resulting isolates was by their growth characteristics on the respective culture media. Potential *S. aureus* and *K. pneumoniae* isolates were also subjected to the coagulase and oxidase tests, respectively. The former was performed with the Bactident^®^ EDTA-rabbit plasma (Merck, Rahway, NJ, USA) according to the manufacturer’s instructions, while the latter was performed with the BactiDrop™ oxidase test reagent (Thermo Fisher Scientific, Lenexa, KS, USA). The chocolate agar cultures were transferred to the STGG medium for long-term storage.

### 2.3. PCR Identification of Target Bacteria from Swab Cultures

For the detection of *S. aureus*, *K. pneumoniae* and *P. aeruginosa*, the potential isolates were subcultured onto LB agar and the resulting cultures were subjected to boiling DNA extraction. The respective DNA extracts were subjected to specific PCR assays targeting the *S. aureus nuc*, *K. pneumoniae mdh* and *P. aeruginosa oprL* genes. For the detection of *S. pneumoniae*, *H. influenzae* and *N. meningitidis*, the suspensions of chocolate agar cultures were subjected to DNA extraction with the GF-1 Tissue DNA Extraction Kit (Vivantis, Selangor, Malaysia) according to the manufacturer’s instructions. The culture DNA extracts were subjected to a multiplex PCR assay targeting the *S. pneumoniae lytA*, *H. influenzae bex* and *N. meningitidis ctrA* genes. The sequences of the primer pairs used in these PCR assays and their respective amplicon sizes are shown in Table 1.

The PCR conditions were adapted from the respective studies [32,33,34,35,36], with some optimizations performed (data not shown). The final conditions used in this study are as follows. Each PCR reaction consisted of 1× PCR buffer (Invitrogen, Carlsbad, CA, USA), 0.3 μM each for forward and reverse primers (Integrated DNA Technologies, Singapore), 0.3 mM dNTPs (RBC Bioscience, New Taipei City, Taiwan), 2.5 mM MgCl_2_ (Invitrogen), 2.5 U of *Taq* DNA polymerase (Invitrogen) and 80-100 ng of template DNA in a total volume of 20 μL. The PCR conditions were 95 °C for 5 min, followed by 35 cycles of 95 °C for 30 s, 57 °C for 40 s and 72 °C for 1 min. A no-template control (NTC) and positive control were included in each run. PCR amplicons were subjected to electrophoresis on 2% (*w*/*v*) agarose gel, which was then stained in RedSafe™ Nucleic Acid Staining Solution (iNtRON Biotechnology, Gyeonggi-do, Korea) for visualization under the ultraviolet transilluminator.

### 2.4. Antibiotic Susceptibility Assessment of ESKAPE Target Bacteria

The Kirby-Bauer antibiotic susceptibility test was performed on the *S. aureus*, *K. pneumoniae* and *P. aeruginosa* isolates, which are three species of the ESKAPE pathogens that are commonly associated with high antibiotic resistance. The bacterial test suspension, with turbidity equivalent to that of the 0.5 McFarland standard, was inoculated over the Mueller–Hinton agar (Oxoid) with a sterile cotton swab to create a bacterial lawn. Antibiotic discs (Oxoid) were placed over the agar and the plates were incubated at 37 °C for 18 h. The results were interpreted by comparing the diameter of the zones of inhibition measured to the Clinical and Laboratory Standards Institute (CLSI) standards [37] to determine the susceptibility of the isolates. *S. aureus* was tested with chloramphenicol (30 μg), ciprofloxacin (5 μg) doxycycline (30 μg), gentamicin (10 μg), methicillin (5 μg), penicillin G (10 U), quinupristin-dalfopristin (15 μg), trimethoprim-sulfamethoxazole (1.25/23.75 μg) and tetracycline (30 μg). *K. pneumoniae* was tested with ampicillin (10 μg), cefpodoxime (10 μg), ceftazidime (30 μg), ceftriaxone (30 μg), ciprofloxacin (5 μg), gentamicin (10 μg), imipenem (10 μg) and tetracycline (30 μg). *P. aeruginosa* was tested with ceftazidime (30 μg), ciprofloxacin (5 μg), gentamicin (10 μg) and imipenem (10 μg). These antibiotics were selected based on the CLSI guidelines, in which many are of the following classes: beta-lactams, aminoglycosides, carbapenems, macrolides, quinolones and tetracyclines.

### 2.5. Data Analysis

Statistical analyses were conducted using the SPSS software version 25.0 (IBM, Armonk, NY, USA). A univariate analysis was performed to determine the distribution of values for all the variables. Fisher’s exact test was used to analyze the associations between demographic characteristics or host factors and bacterial carriage. A *p*-value of less than 0.05 would indicate statistical significance at a 95% confidence level.

## 3. Results

### 3.1. Subject Demographic and Health-Related Profiles

A total of 100 subjects participated in this study, in which 100 nasal and 100 oropharyngeal swabs were collected and tested. The median age of the subjects was 21 years old, with an equal number in gender distribution. The ethnicity distribution is as follows: 92 Chinese, 5 Indian and 3 Malay. This skewed distribution mirrored the ethnic composition of the student population at the university’s main campus (the study location) during the study period: 92% Chinese (*n* = 12,184), 6.2% Indian (*n* = 828), 0.6% Malay (*n* = 75) and 1.5% Others (*n* = 193). Based on eligibility for receiving vaccines under the Malaysian National Immunisation Programme (NIP), only 27% of the subjects were aware of their vaccination status, while 55% were unsure and the remaining thought they did not receive any of the vaccines. Thirty-five subjects reported having URT symptoms that could be those of respiratory infections (e.g., cold, flu, ear infection, etc.) in the month prior to sample collection. Five subjects had taken antibiotics in this period.

### 3.2. Carriage Prevalence of Target Bacteria

**Figure 1:**
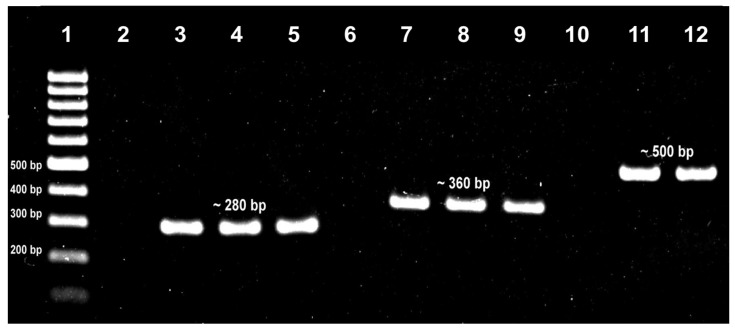
Gel analysis for singleplex PCR assays. Lane 1: 100-bp DNA ladder; Lane 2: *nuc* PCR NTC; Lane 3: *nuc* PCR positive control (*S. aureus* ATCC 6538), Lanes 4–5: *nuc* PCR test isolates; Lane 6: *mdh* PCR NTC; Lane 7: *mdh* PCR positive control (*K. pneumoniae* ATCC 13883), Lanes 8–9: *mdh* PCR test isolates; Lane 10: *oprL* PCR NTC; Lane 11: *oprL* PCR positive control (*P. aeruginosa* ATCC 9027), Lane 12: *oprL* PCR test isolate.

**Figure 2:**
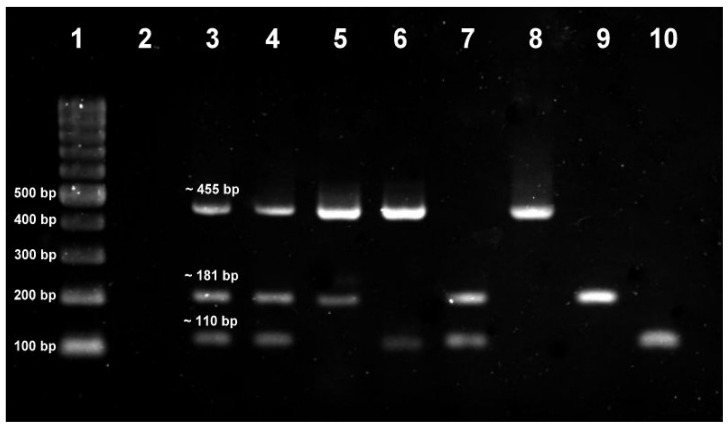
Gel analysis for multiplex PCR assay targeting *S. pneumoniae*, *H. influenzae* and *N. meningitidis*. Lane 1: 100-bp DNA ladder; Lane 2: NTC; Lane 3: positive control: *S. pneumoniae* ATCC 6303 *lytA* (455 bp), *H. influenzae* ATCC 10211 *bex* (181 bp) and *N. meningitidis* ATCC 10377 *ctrA* (110 bp); Lane 4: sample with all three target bacteria; Lane 5: sample with *S. pneumoniae* and *H. influenzae*; Lane 6: sample with *S. pneumoniae* and *N. meningitidis*; Lane 7: sample with *H. influenzae* and *N. meningitidis*; Lane 8: sample with only *S. pneumoniae*; Lane 9: sample with only *H. influenzae*; Lane 10: sample with only *N. meningitidis*.

Potential isolates of *S. aureus*, *K. pneumoniae* and *P. aeruginosa* from swab cultures on mannitol salt agar (yellow colonies with yellow zones), MacConkey agar (pink mucoid colonies) and Pseudomonas agar supplemented with C-N (green colonies), respectively, were subjected to their respective PCR assays. Based on the PCR results, the carriage prevalence among the 100 subjects was 27%, 11% and 1%, respectively. Figure 1 shows the representative gel analysis for the *S. aureus nuc*, *K. pneumoniae mdh* and *P. aeruginosa oprL* PCR assays. All the potential *S. aureus* isolates were coagulase-positive, while all the *K. pneumoniae* isolates were oxidase-negative. The detection of *S. pneumoniae*, *H. influenzae* and *N. meningitidis* in the swab cultures on chocolate agar was by a multiplex PCR assay on their total DNA extracts. Based on the PCR results, the carriage prevalence among the 100 subjects was 15%, 36% and 5%, respectively. Figure 2 shows the representative gel analysis for the multiplex PCR assay.

### 3.3. Bacterial Carriage by URT Sites

The distribution of target bacterial carriage between the two URT sites surveyed, the anterior nares and the oropharynx, was also determined. From a total of 31 *S. aureus* isolates obtained from 27 subjects, 21 (67.7%) were from the nasal swabs and 10 (32.3%) were from the oropharyngeal swabs. Four subjects were positive for *S. aureus* by both swabs. For the 12 *K. pneumoniae* isolates obtained from 11 subjects, 5 (41.7%) were of nasal origin and 7 (58.3%) were from the oropharynx. The sole *P. aeruginosa* in this study was isolated from the oropharyngeal swab. For *S. pneumoniae*, 13 of 15 subjects (86.7%) carried it in the oropharynx while the remaining two subjects (13.3%) showed nasal carriage. All the *H. influenzae* (*n* = 36) and *N. meningitidis* (*n* = 5) carriage was in the oropharynx.

### 3.4. Bacterial Co-Carriage

**Figure 3:**
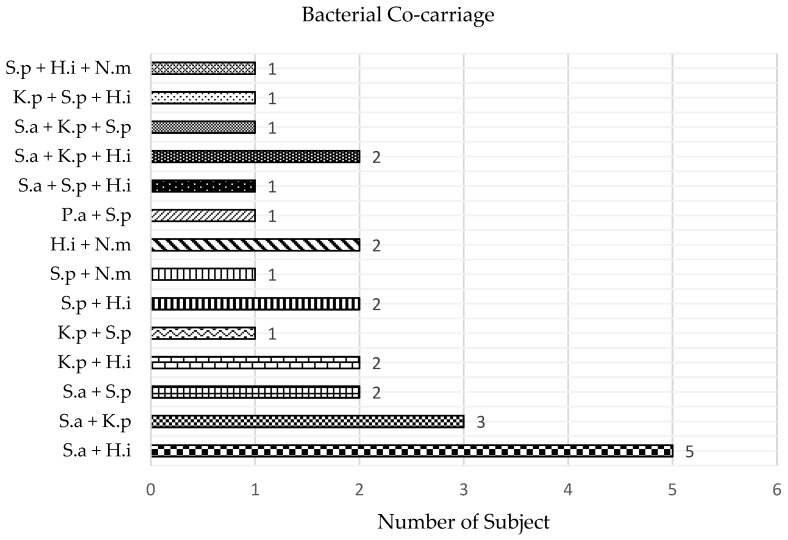
Bacterial co-carriage among the subjects. S.a—*S. aureus*; K.p—*K. pneumoniae*; P.a—*P. aeruginosa*; S.p—*S. pneumoniae*; H.i—*H. influenzae*; N.m—*N. meningitidis*.

Co-carriage prevalence was determined by the proportions of subjects carrying more than one target bacterium. Of the 64 carriers in this study, 25 belonged to this category, of which 19 subjects (29.7%) carried two target bacteria and 6 subjects (9.4%) carried three target bacteria. Figure 3 shows that the co-carriage of *S. aureus* and *H. influenzae* was the highest (*ո* = 5), followed by that of *S. aureus* and *K. pneumoniae* (*ո* = 3). Other co-carriage combinations were observed in 1–2 subjects (Figure 3). For the 39 subjects with single bacterial carriage, there were 20 (31.3%) *H. influenzae* carriers, 13 (20.3%) *S. aureus* carriers, 4 (6.3%) *S. pneumoniae* carriers and one carrier (1.6%) each for *K. pneumoniae* and *N. meningitidis*.

### 3.5. Association between Bacterial Carriage and Host Factors

As shown in Table 2, the overall carriage of target bacteria was significantly higher in males (*n* = 39; 78%) as compared to in females (*n* = 25; 50%) (*p* < 0.01). Additionally, males showed a higher frequency of multiple bacterial carriage (≥2 target bacteria) as compared to females (*p* < 0.01). No significant associations were demonstrated between the bacterial carriage and the other factors (ethnicity, NIP vaccination, previous URT symptoms and antibiotic intake). When comparing the URT sites among the target bacterial carriers, there are no significant differences for all the factors (data not shown). It is noteworthy that the assessment for some of these factors was limited by skewed distribution in their variables, which is especially apparent for ethnicity and antibiotic intake. The reliability of responses to NIP vaccination is another concern.

### 3.6. Antibiotic Susceptibility of ESKAPE Target Bacteria

A high level of penicillin resistance (51.6%, *n* = 16) was observed among the *S. aureus* isolates (Table 3). For quinupristin-dalfopristin, 2 (6.5%) isolates were resistant to it, but 8 (25.8%) isolates fall into the category of intermediate resistance. Resistance or intermediate resistance to chloramphenicol, doxycycline, gentamicin, methicillin and tetracycline was also occasionally observed in some isolates. All the *S. aureus* isolates were susceptible to ciprofloxacin and trimethoprim/sulfamethoxazole. For *K. pneumoniae*, all the isolates were resistant to ampicillin but susceptible to the other seven antibiotics (Table 3). The only *P. aeruginosa* isolate from this study was susceptible to all four antibiotics tested, which were ceftazidime, ciprofloxacin, gentamicin and imipenem. None of the bacterial isolates showed multidrug resistance, which is commonly defined as resistance to antibiotics of three or more classes [38].

## 4. Discussion

Based on the results obtained, the carriage prevalence of the target bacteria among the university students (*n* = 100) was as follows: *S. pneumoniae* (15%), *H. influenzae* (36%), *N. meningitidis* (5%), *S. aureus* (27%), *K. pneumoniae* (11%) and *P. aeruginosa* (1%). In two Nepalese studies on undergraduate medical students and healthcare workers, the *S. pneumoniae* carriage prevalence was 4% and 21%, respectively [20,39]. The pneumococcal carriage was also documented to be relatively low (3.2%) among medical students in a Turkish study [40]. A recent study among the indigenous community (70.7% adults) in Malaysian Borneo and another on adults in Indonesia recorded carriage prevalence of 10% and 11%, respectively [28,41]. With regard to *S. aureus* carriage, several studies reported a prevalence of 20–30% [28,42,43], which is consistent with that observed in our study. Nevertheless, two studies among students of two Malaysian universities recorded a lower prevalence of 10% and 16%, respectively [22,23]. For *K. pneumoniae* carriage, the prevalence in our study (11%) is identical to that documented among adults in an Indonesian study [41]. The Turkish study on medical students reported a significantly lower carriage prevalence of 0.8% [40], while the Borneo study reported a much higher prevalence of 30% among their target indigenous community in rural East Malaysia [28].

Meningococcal carriage is relatively more common in late adolescents and young adults [44]. A study on South Australian university students showed a carriage prevalence of 6.2% [44], which is comparable to the prevalence (5%) in our study. The Borneo study also recorded a prevalence of 5% [28]. However, another study reported a higher carriage prevalence of 12.7–14.6% among undergraduate students in a university in the USA [45]. In two carriage studies in Turkey, a low prevalence of *N. meningitidis* carriage was observed among medical students and the community, both at 0.6% [40,46]. For *P. aeruginosa* carriage, only one subject (1%) in our study was positive, consistent with the low carriage prevalence reported among medical students in the Turkish study [40]. *P. aeruginosa* is a nosocomial pathogen that seldom circulates in the healthy population, which might explain its low carriage in the community. However, a significantly higher prevalence of 6.4% was reported among the indigenous population in the Borneo study [28].

Intriguingly, the *H. influenzae* carriage was unexpectedly high in our study as compared to that reported by studies in other countries. The Nepalese studies documented carriage of 12.5% and 8% for *H. influenzae* among undergraduate medical students and healthcare workers, respectively [20,39]. The *H. influenzae* carriage among medical students in the Turkish study was also observed less frequently, with a prevalence of 3.2% [40], while the Borneo study recorded a prevalence of 9.3% among the indigenous community [28]. The *Haemophilus* type b (Hib) conjugate vaccine was added to the Malaysian NIP in 2002; it is given to children at 2, 3 and 5 months of age [47]. Despite its implementation, the effects of Hib vaccination have yet to be sufficiently evaluated and monitored. It is noteworthy that all our subjects were born before 2002 and might not benefit from certain protective effects of the Hib vaccine. The possibility of cross-detection of closely related *Haemophilus* species or strains by the PCR used cannot be excluded at this stage, though in silico analysis has demonstrated its specificity for capsulated *H. influenzae* [48].

The distribution of target bacterial carriage in the URT sites surveyed was also determined. Most of the *S. aureus* isolates in this study (21 of 31 isolates, 67.7%) and other carriage studies were recovered from the anterior nares in line with its predominance in the nasal microbiome [14]. For *S. pneumoniae* and *H. influenzae*, 13 of 15 subjects (86.7%) and all 36 subjects (100%) carried them in the oropharynx, respectively. Similar to *H. influenzae*, all 5 subjects (100%) that were positive for *N. meningitidis* carried it in the oropharynx. The common presence of these three bacteria in the oropharynx is often reported [10,15]. Though *K. pneumoniae* carriage is commonly reported in the oropharynx [28], our findings show significant nasal carriage among the subjects. This illustrates the difference in microbial profile among the URT sites that would influence target bacterial detection by the sampling methods used. Individual variation in this is another factor to be considered.

Interactions between different species (also known as polymicrobial interactions) in URT carriage may be key to understanding their role in the occurrence of lower respiratory tract infection [11,18]. One of the most common interactions documented is between *S. pneumoniae* and *H. influenzae* [11,13,49]. In our study, the highest co-carriage was that between *S. aureus* and *H. influenzae*. Similarly, a study demonstrated that levels of *H. influenzae* were higher when *S. aureus* was the initial colonizer in the murine nasopharynx [50]. However, one study reported a negative association between *S. aureus* and *H. influenzae* for carriage in children, thus suggesting their competition for URT colonization [18]. Though some subjects in this study showed *S. aureus* and *S. pneumoniae* co-carriage, several studies reported their negative association due to the inflammatory and humoral immune responses triggered by the latter [11,18]. The low co-carriage prevalence in this study is expected to limit the interpretation of their significance.

The male subjects in this study showed significantly higher bacterial carriage relative to the female subjects. Several carriage studies have reported this observation, in which two demonstrated higher carriage of *S. aureus* and methicillin-resistant *S. aureus* (MRSA) among males [20,42]. Available data also point to males being more susceptible to respiratory and other infections and likely to develop more severe infections than females [51,52,53]. These could be supported by the effects of hormonal differences, with the female hormone estrogen assumed to have immune-stimulating properties at physiological concentrations, while the male hormone testosterone is often associated with immune suppression [51,52,54]. There is strong evidence that postmenopausal women with undetectable plasma estrogen have a reduced adaptive immune response [55]. Despite their asymptomatic presence, the interplay between mucosal immunity and URT colonization by pathogens is generally known [15]. One study showed the production of salivary antibodies against the capsular polysaccharides and surface-associated proteins of *S. pneumoniae* in response to its URT colonization [56]. Another consideration for the gender factor is how differences in behavioral practices, such as hand hygiene, would influence bacterial colonization and infection rates [53,57,58]. Nevertheless, one study on the indigenous population and another on university students in Malaysia showed that *S. aureus* carriage was more prevalent among females [23,27].

No significant associations were demonstrated for the other host factors (ethnicity, NIP vaccination, previous URT symptoms and antibiotic intake) in this study. The NIP aims to increase national immunization coverage to help reduce vaccine-preventable diseases, in which it provides free vaccines to protect those eligible against these diseases. Under the Malaysian NIP, 13 vaccines are provided for protection against major childhood diseases, with the pneumococcal conjugate vaccine added in December 2020 [59,60]. The latter highlights the need for respiratory carriage studies as surveillance and to evaluate its success. In the questionnaire survey, more than half of our subjects (55%) were unsure if they received any of the vaccines provided under the NIP, while 18% thought they never received any. This is of concern and reflects a lack of awareness and knowledge in this health aspect, even among the educated segment of the population. A KAP (knowledge, attitude and practice) survey on this and other health aspects among the target population is warranted to complement the existing carriage studies.

The antibiotic susceptibility of *S. aureus*, *K. pneumoniae* and *P. aeruginosa* was emphasized in this study due to them being members of the ESKAPE family, which is commonly associated with increased ability to acquire antibiotic resistance and the emergence of multidrug resistance [61]. The term “ESKAPE” is an acronym for scientific names of six bacterial pathogens, which include *Enterococcus faecium*, *S. aureus*, *K. pneumoniae*, *Acinetobacter baumannii*, *P. aeruginosa* and *Enterobacter* spp. Their presence is of concern to community spread of multidrug-resistant strains and the use of antibiotic prophylaxis to reduce URT bacterial carriage [62]. The high rate of penicillin resistance among the *S. aureus* isolates in our study (51.6%) was in concordance with another study performed on students in another local university (49%) [43]. Widespread penicillin usage rapidly resulted in the emergence of 𝛽-lactamase-producing *Staphylococcus* strains, with 65–85% of circulating *S. aureus* showing resistance to penicillin G [63]. All the *K. pneumoniae* isolates and the sole *P. aeruginosa* isolate obtained were susceptible to all the antibiotics tested, except for ampicillin for the former. *Klebsiella* species are known to be intrinsically resistant to ampicillin due to the production of SHV-1 beta-lactamase; this can be used as a marker for the identification of *K. pneumoniae* [64,65]. The inclusion of some third-generation cephalosporins in the test was to screen for the potential presence of extended-spectrum beta-lactamase (ESBL) *K. pneumoniae* strains. Even though published clinical data for antimicrobial resistance has increased, there is a scarcity of this information among carriage isolates and their surveillance [28]. Bacterial carriage has been recognized as a potential source of antibiotic-resistant pathogens, especially among those that practice self-medication with antibiotics from local retail pharmacies. One study reported this practice among students at a Malaysian university in which penicillin was one of the most commonly abused antibiotics [66].

## 5. Conclusions

The findings of this study are of value in providing important insights into the epidemiology and drug susceptibility profiles of the bacterial pathogens commonly associated with URT carriage, studies of which are currently still limited in the Malaysian population. Continued surveillance of URT carriage in the community will provide a better understanding of the risk of disease acquisition from bacterial carriage and help to evaluate the effectiveness of current and future infection control practices at the population level.

## Figures and Tables

**Table 1:** Sequences of primer pairs used in the PCR assays.

Target Bacterium	Target Gene	Primer Sequences (5′ → 3′)	Amplicon Size	Reference
*S. aureus*	*nuc*	Forward: GCGATTGATGGTGATACGGTT Reverse: AGCCAAGCCTTGGAACTAAAGC	279 bp	[32]
*K. pneumoniae*	*mdh*	Forward: GCGTGGCGGTAGATCTAAGTCATA Reverse: TTCAGCTCCGCCACAAAGGTA	364 bp	[33]
*P. aeruginosa*	*oprL*	Forward: ATGGAAATGCTGAAATTCGGC Reverse: CTTCTTCAGCTCGACGCGACG	504 bp	[34]
*S. pneumoniae*	*lytA*	Forward: TGCCTCAAGTCGGCGTGCAA Reverse: CTGCTCACGGCTAATGCCCCAT	455 bp	[35]
*H. influenzae*	*bex*	Forward: TATCACACAAATAGCGGTTGG Reverse: GGCCAAGAGATACTCATAGAACG	181 bp	[36]
*N. meningitidis*	*ctrA*	Forward: GCTGCGGTAGGTGGTTCAA Reverse: TTGTCGCGGATTTGCAACTA	110 bp	[36]

**Table 2:** Bacterial carriage and its association with host factors.

Host Factor	Overall Bacterial Carriage		Bacterial Carriage, *n* (%)		
	*n* (%)	*p*-value ^a^	Single sp.	Multiple spp.	*p*-value ^b^
**Gender**					
Male	39 (78)	0.006 *	21 (42)	18 (36)	0.005 *
Female	25 (50)		18 (36)	7 (14)	
**Ethnicity**					
Malay	2 (66.7)	0.99	2 (66.7)	0 (0)	0.96
Chinese	59 (64.1)		35 (38)	24 (26.1)	
Indian	3 (60)		2 (40)	1 (20)	
**NIP Vaccination**					
Yes	18 (66.7)	0.88	11 (40.7)	7 (25.9)	0.99
No	12 (66.7)		7 (38.9)	5 (27.8)	
Unsure	34 (61.8)		21 (38.2)	13 (23.6)	
**Previous URT Symptoms**					
Yes	24 (68.6)	0.52	13 (37.1)	11 (31.4)	0.56
No	40 (61.5)		26 (40)	14 (21.5)	
**Previous Antibiotic Intake**					
Yes	2 (40)	0.35	1 (20)	1 (20)	0.53
No	62 (65.3)		38 (40)	24 (25.3)	

^a^ Between group comparison for overall bacterial carriage by Fisher’s exact test. ^b^ Between group comparison for multiple bacterial carriage by Fisher’s exact test. * *p* < 0.05.

**Table 3:** Antibiotic susceptibility profiles of the ESKAPE target isolates.

Antibiotics	Susceptibility		
	Susceptible	Intermediate	Resistant
***S. aureus*** (*n* = 31)			
Chloramphenicol	29 (93.5%)	2 (6.5%)	0 (0%)
Ciprofloxacin	31 (100%)	0 (0%)	0 (0%)
Doxycycline	30 (96.8%)	0 (0%)	1 (3.2%)
Gentamicin	30 (96.8%)	1 (3.2%)	0 (0%)
Methicillin	30 (96.8%)	0 (0%)	1 (3.2%)
Penicillin G	15 (48.4%)	0 (0%)	16 (51.6%)
Quinupristin-dalfopristin	21 (67.7%)	8 (25.8%)	2 (6.5%)
Trimethoprim/sulfamethoxazole	31 (100%)	0 (0%)	0 (0%)
Tetracycline	30 (96.8%)	0 (0%)	1 (3.2%)
***K. pneumoniae*** (*n* = 12)			
Ampicillin	0 (0%)	0 (0%)	12 (100%)
Chloramphenicol	12 (100%)	0 (0%)	0 (0%)
Cefpodoxime	12 (100%)	0 (0%)	0 (0%)
Ceftazidine	12 (100%)	0 (0%)	0 (0%)
Ceftriaxone	12 (100%)	0 (0%)	0 (0%)
Gentamicin	12 (100%)	0 (0%)	0 (0%)
Imipenem	12 (100%)	0 (0%)	0 (0%)
Tetracycline	12 (100%)	0 (0%)	0 (0%)
***P. aeruginosa*** (*n* = 1)			
Ceftazidime	1 (100%)	0 (0%)	0 (0%)
Ciprofloxacin	1 (100%)	0 (0%)	0 (0%)
Gentamicin	1 (100%)	0 (0%)	0 (0%)
Imipenem	1 (100%)	0 (0%)	0 (0%)

## Data Availability

All data generated or analyzed during this study are included in this published article.

## References

[1] García-Rodríguez J.Á., Martínez M.J.F. (2002). Dynamics of nasopharyngeal colonization by potential respiratory pathogens. J. Antimicrob. Chemother..

[2] Kumpitsch C., Koskinen K., Schöpf V., Moissl-Eichinger C. (2019). The microbiome of the upper respiratory tract in health and disease. BMC Biol..

[3] Cleary D.W., Clarke S.C. (2017). The nasopharyngeal microbiome. Emerg. Top. Life Sci..

[4] Ghssein G., Ezzeddine Z. (2022). The key element role of metallophores in the pathogenicity and virulence of *Staphylococcus aureus*: A Review. Biology.

[5] Ghssein G., Ezzeddine Z. (2022). A review of *Pseudomonas aeruginosa* metallophores: Pyoverdine, pyochelin and pseudopaline. Biology.

[6] Tsang R.S.W. (2021). A narrative review of the molecular epidemiology and laboratory surveillance of vaccine preventable bacterial meningitis agents: *Streptococcus pneumoniae*, *Neisseria meningitidis*, *Haemophilus influenzae* and *Streptococcus agalactiae*. Microorganisms.

[7] Martin R.M., Bachman M.A. (2018). Colonization, infection, and the accessory genome of *Klebsiella pneumoniae*. Front. Cell Infect. Microbiol..

[8] Clark S.C. (2020). Commensal bacteria in the upper respiratory tract regulate susceptibility to infection. Curr. Opin. Immunol..

[9] Haak B.W., Brands X., Davids M., Peters-Sengers H., Kullberg R.F.J., van Houdt R., Hugenholtz F., Faber D.R., Zaaijer H.L., Scicluna B.P. (2022). Bacterial and viral respiratory tract microbiota and host characteristics in adults with lower respiratory tract infections: A case-control study. Clin. Infect. Dis..

[10] Feldman C., Anderson R. (2019). Meningococcal pneumonia: A review. Pneumonia.

[11] Claassen-Weitz S., Lim K.Y.L., Mullally C., Zar H.J., Nicol M.P. (2021). The association between bacteria colonizing the upper respiratory tract and lower respiratory tract infection in young children: A systematic review and meta-analysis. Clin. Microbiol. Infect..

[12] Subramanian K., Henriques-Normark B., Normark S. (2019). Emerging concepts in the pathogenesis of the *Streptococcus pneumoniae*: From nasopharyngeal colonizer to intracellular pathogen. Cell Microbiol..

[13] Coughtrie A.L., Whittaker R.N., Begum N., Anderson R., Tuck A., Faust S., Jefferies J.M., Yuen H.M., Roderick P.J., A Mullee M. (2014). Evaluation of swabbing methods for estimating the prevalence of bacterial carriage in the upper respiratory tract: A cross sectional study. BMJ Open.

[14] Brown A.F., Leech J.M., Rogers T.R., McLoughlin R.M. (2014). *Staphylococcus aureus* colonization: Modulation of host immune response and impact on human vaccine design. Front. Immunol..

[15] Bogaert D., de Groot R., Hermans P.W.M. (2004). *Streptococcus pneumoniae* colonisation: The key to pneumococcal disease. Lancet Infect. Dis..

[16] Christensen H., May M., Bowen L., Hickman M., Trotter C. (2010). Meningococcal carriage by age: A systematic review and meta-analysis. Lancet Infect. Dis..

[17] Schenck L.P., Surette M.G., Bowdish D.M. (2016). Composition and immunological significance of the upper respiratory tract microbiota. FEBS Lett..

[18] Pettigrew M.M., Gent J.F., Revai K., Patel J.A., Chonmaitree T. (2008). Microbial interactions during upper respiratory tract infections. Emerg. Infect. Dis..

[19] McNeil H.C., Clarke S.C. (2016). Serotype prevalence of *Streptococcus pneumoniae* in Malaysia—The need for carriage studies. Med. J. Malays..

[20] Bhatta D.R., Hamal D., Shrestha R., Parajuli R., Baral N., Subramanya S.H., Nayak N., Gokhale S. (2018). Nasal and pharyngeal colonization by bacterial pathogens: A comparative study between preclinical and clinical sciences medical students. Can. J. Infect. Dis. Med. Microbiol..

[21] Peterson M.E., Mile R., Li Y., Nair H., Kyaw M.H. (2018). Meningococcal carriage in high-risk settings: A systematic review. Int. J. Infect. Dis..

[22] Nordin S.A., Za’im N.A.N., Sahari N.N., Jamaluddin S.F., Ahmadi S., Desa M.N.M. (2012). *Staphylococcus aureus* nasal carriers among medical students in a medical school. Med. J. Malays..

[23] Zuridah H., Wirdatul N.M.K., Rashidah S., Evana K., Ahmad N.M.R., Siti A.B., Zakri A.H.Z., Izham N.S.A.S. (2011). Carriage patterns and susceptibility testing of *Staphylococcus aureus* in healthy nasal carriers in UiTM. Biohealth Sci. Bull..

[24] Fong I.L., Abdul R.E.E., Tham J.J.M., Safian N.A., Ong S.T., Ng P.P., Hazmi H. (2018). Prevalence and antibiotic sensitivity profiles of *Staphylococcus aureus* nasal carriage among preclinical and clinical medical students in a Malaysian university. Malays. J. Microbiol..

[25] Le C.F., Jefferies J.M., Yusof M.Y.M., Sekaran S.D., Clarke S. (2012). The epidemiology of pneumococcal carriage and infections in Malaysia. Expert Rev. Anti-Infect. Ther..

[26] Malik A.S., Ismail A., Pennie R.A., Naidu J.V. (1998). Susceptibility pattern of *Streptococcus pneumoniae* among pre-school children in Kota Bharu, Malaysia. J. Trop. Pediatr..

[27] Cleary D.W., Morris D.E., Anderson R.A., Jones J., Alattraqchi A.G., Rahman N.I.A., Ismail S., Razali M.S., Amin R.M., Aziz A.A. (2021). The upper respiratory tract microbiome of indigenous Orang Asli in north-eastern Peninsular Malaysia. npj Biofilms Microbiomes.

[28] Morris D.E., McNeil H., Hocknell R.E., Anderson R., Tuck A.C., Tricarico S., Norazmi M.N., Lim V., Siang T.C., on behalf of the MYCarriage group (2021). Carriage of upper respiratory tract pathogens in rural communities of Sarawak, Malaysian Borneo. Pneumonia.

[29] Lister A.J.J., Le C.F., Cheah E.S.G., Desa M.N.M., Cleary D.W., Clarke S.C. (2021). Serotype distribution of invasive, non-invasive and carried *Streptococcus pneumoniae* in Malaysia: A meta-analysis. Pneumonia.

[30] Dean A.G., Arner T.G., Sunki G.G., Friedman R., Lantinga M., Sangam S., Zubieta J.C., Sullivan K.M., Brendel K.A., Gao Z. (2011). Epi Info™, Version 7.2.2.6.

[31] Satzke C., Turner P., Virolainen-Julkunen A., Adrian P.V., Antonio M., Hare K.M., Henao-Restrepo A.M., Leach A.J., Klugman K.P., Porter B.D. (2013). Standard method for detecting upper respiratory carriage of *Streptococcus pneumoniae*: Updated recommendations from the World Health Organization Pneumococcal Carriage Working Group. Vaccine.

[32] Xirogianni A., Tzanakaki G., Karagianni E., Markoulatos P., Kourea-Kremastinou J. (2009). Development of a single-tube polymerase chain reaction assay for the simultaneous detection of *Haemophilus influenzae, Pseudomonas aeruginosa, Staphylococcus aureus*, and *Streptococcus spp*. directly in clinical samples. Diagn. Microbiol. Infect. Dis..

[33] Thong K.L., Lai M.Y., Teh C.S.J., Chua K.H. (2011). Simultaneous detection of methicillin-resistant *Staphylococcus aureus*, *Acinetobacter baumannii*, *Escherichia coli*, *Klebsiella pneumoniae* and *Pseudomonas aeruginosa* by multiplex PCR. Trop. Biomed..

[34] Jiang L.X., Ren H.Y., Zhou H.J., Zhao S.H., Hou B.Y., Yan J.P., Qin T., Chen Y. (2017). Simultaneous detection of 13 key bacterial respiratory pathogens by combination of multiplex PCR and capillary electrophoresis. Biomed. Environ. Sci..

[35] Lee C.-T., Hsiao K.-M., Chen J.-C., Su C.-C. (2015). Multiplex polymerase chain reaction assay developed to diagnose adult bacterial meningitis in Taiwan. APMIS.

[36] Tzanakaki G., Tsopanomichalou M., Kesanopoulos K., Matzourani R., Sioumala M., Tabaki A., Kremastinou J. (2005). Simultaneous single-tube PCR assay for the detection of *Neisseria meningitidis*, *Haemophilus influenzae* type b and *Streptococcus pneumoniae*. Clin. Microbiol. Infect..

[37] CLSI (2020). Performance Standards for Antimicrobial Susceptibility Testing. CLSI Supplement M100.

[38] Magiorakos A.-P., Srinivasan A., Carey R.B., Carmeli Y., Falagas M.E., Giske C.G., Harbarth S., Hindler J.F., Kahlmeter G., Olsson-Liljequist B. (2012). Multidrug-resistant, extensively drug-resistant and pandrug-resistant bacteria: An international expert proposal for interim standard definitions for acquired resistance. Clin. Microbiol. Infect..

[39] Hosuru Subramanya S., Thapa S., Dwedi S.K., Gokhale S., Sathian B., Nayak N., Bairy I. (2016). *Streptococcus pneumoniae* and *Haemophilus* species colonization in health care workers: The launch of invasive infections?. BMC Res. Notes..

[40] Karapinar B.A., Yürüyen C., Gürler N., Kayacan C. (2019). Nasopharyngeal carriage of *Neisseria meningitidis* among medical school students in Turkey. Biomed Res..

[41] Farida H., Severin J.A., Gasem M.H., Keuter M., van den Broek P., Hermans P.W., Wahyono H., Verbrugh H.A. (2013). Nasopharyngeal carriage of *Klebsiella pneumoniae* and other Gram-negative bacilli in pneumonia-prone age groups in Semarang, Indonesia. J. Clin. Microbiol..

[42] Humphreys H., Fitzpatick F., Harvey B.J. (2015). Gender differences in rates of carriage and bloodstream infection caused by methicillin-resistant *Staphylococcus aureus*: Are they real, do they matter and why?. Clin. Infect. Dis..

[43] Suhaili Z., Rafee P., Mat Azis N., Yeo C.C., Nordin S.A., Abdul Rahim A.R., Al-Obaidi M.M.J., Mohd Desa M.N. (2018). Characterization of resistance to selected antibiotics and Panton-Valentine leukocidin-positive *Staphylococcus aureus* in a healthy student population at a Malaysian University. Germs.

[44] McMillan M., Walters L., Mark T., Lawrence A., Leong L.E.X., Sullivan T., Rogers G.B., Andrews R.M., Marshall H.S. (2018). B part of it study: A longitudinal study to assess carriage of *Neisseria meningitidis* in first year university students in South Australia. Hum. Vaccin Immunother..

[45] Breakwell L., Whaley M., Khan U.I., Bandy U., Alexander-Scott N., Dupont L., Vanner C., Chang H.-Y., Vuong J.T., Martin S. (2018). Meningococcal carriage among a university student population–United States, 2015. Vaccine.

[46] Kepenekli Kadayifci E., Güneşer Merdan D., Soysal A., Karaaslan A., Atıcı S., Durmaz R., Boran P., Turan İ., Söyletir G., Bakır M. (2016). Prevalence of *Neisseria meningitidis* carriage: A small-scale survey in Istanbul, Turkey. J. Infect. Dev. Ctries..

[47] (2019). Ministry of Health Malaysia, Health Facts. http://www.moh.gov.my/moh/resources/Penerbitan/Penerbitan%20Utama/HEALTH%20FACTS/Healh%20Facts%202019_Booklet.pdf.

[48] Torigoe H., Seki M., Yamashita Y., Sugaya A., Maeno M. (2007). Detection of *Haemophilus influenzae* by loop-mediated isothermal amplification (LAMP) of the outer membrane protein P6 gene. Jpn. J. Infect. Dis..

[49] Cleary D., Devine V., Morris D., Osman K., Gladstone R., Bentley S., Faust S., Clarke S. (2018). Pneumococcal vaccine impacts on the population genomics of non-typeable *Haemophilus influenzae*. Microb. Genom..

[50] Margolis E., Yates A., Levin B.R. (2010). The ecology of nasal colonization of *Streptococcus pneumoniae*, *Haemophilus influenzae* and *Staphylococcus aureus*: The role of competition and interactions with host’s immune response. BMC Microbiol..

[51] Falagas M.E., Mourtzoukou E.G., Vardakas K.Z. (2007). Sex differences in the incidence and severity of respiratory tract infections. Respir. Med..

[52] Vázquez-Martínez E.R., García-Gómez E., Camacho-Arroyo I., González-Pedrajo B. (2018). Sexual dimorphism in bacterial infections. Biol. Sex Differ..

[53] Ibrahim J.N., Eghnatios E., El Roz A., Fardoun T., Ghssein G. (2019). Prevalence, antimicrobial resistance and risk factors for campylobacteriosis in Lebanon. J. Infect. Dev. Ctries..

[54] Trigunaite A., Dimo J., Jørgensen T.N. (2015). Suppressive effects of androgens on the immune system. Cell. Immunol..

[55] Kamada M., Irahara M., Maegawa M., Yasui T., Takeji T., Yamada M., Tezuka M., Kasai Y., Aono T. (2000). Effect of hormone replacement therapy on post-menopausal changes of lymphocytes and T cell subsets. J. Endocrinol. Investig..

[56] Simell B., Kilpi T.M., Käyhty H. (2002). Pneumococcal carriage and otitis media induce salivary antibodies to pneumococcal capsular polysaccharides in children. J. Infect. Dis..

[57] Garbutt C., Simmons G., Patrick D., Miller T. (2007). The public hand hygiene practices of New Zealanders: A national survey. New Zealand Med. J..

[58] Mackert M., Liang M.C., Champlin S. (2013). “Think the sink:” Preliminary evaluation of a handwashing promotion campaign. Am. J. Infect. Control.

[59] Immunise4Life The Malaysian National Immunisation Programme (NIP). https://immunise4life.my/the-malaysian-national-immunisation-programme-nip/.

[60] CodeBlue Malaysia Chooses PCV10 Pneumococcal Vaccine, Immunisation Starts December. https://codeblue.galencentre.org/2020/11/24/malaysia-chooses-pcv10-pneumococcal-vaccine-immunisation-starts-december/.

[61] WHO (2017). Prioritization of Pathogens to Guide Discovery, Research and Development of New Antibiotics for Drug-Resistant Bacterial Infections, Including Tuberculosis.

[62] Tacconelli E., Carrara E., Savoldi A., Harbarth S., Mendelson M., Monnet D.L., Pulcini C., Kahlmeter G., Kluytmans J., Carmeli Y. (2018). Discovery, research, and development of new antibiotics: The WHO priority list of antibiotic-resistant bacteria and tuberculosis. Lancet Infect. Dis..

[63] Cheng M.P., René P., Cheng A.P., Lee T.C. (2016). Back to the future: Penicillin-susceptible *Staphylococcus aureus*. Am. J. Med..

[64] Wyres K.L., Holt K.E. (2018). *Klebsiella pneumoniae* as a key trafficker of drug resistance genes from environmental to clinically important bacteria. Curr. Opin. Microbiol..

[65] Shaikh S., Fatima J., Shakil S., Rizvi S.M.D., Kamal M.A. (2015). Antibiotic resistance and extended spectrum beta-lactamases: Types, epidemiology and treatment. Saudi J. Biol. Sci..

[66] Haque M., A Rahman N.A., McKimm J., Kibria G.M., Majumder A.A., Haque S.Z., Islam Z., Abdullah S.L.B., Daher A.M., Zulkifli Z. (2019). Self-medication of antibiotics: Investigating practice among university students at the Malaysian National Defence University. Infect. Drug Resist..

